# The impact of COVID-19 on clinical outcomes in people undergoing neurosurgery: a systematic review and meta-analysis

**DOI:** 10.1186/s13643-023-02291-5

**Published:** 2023-08-07

**Authors:** Haiying Teng, Zilan Wang, Xingyu Yang, Xiaoxiao Wu, Zhouqing Chen, Zhong Wang, Gang Chen

**Affiliations:** 1 Department of Neurosurgery & Brain and Nerve Research Laboratory, The First Affiliated Hospital of Soochow University, 188 Shizi Street, Suzhou, 215006 Jiangsu Province China; 2 Suzhou Medical College of Soochow University, Suzhou, 215002 Jiangsu Province China

**Keywords:** Cerebrovascular disease, COVID-19, Neuro-oncology, Neurosurgery, Systematic review

## Abstract

**Background:**

The coronavirus disease-2019 (COVID-19) pandemic has created a global crisis unique to the healthcare system around the world. It also had a profound impact on the management of neurosurgical patients. In our research, we investigated the effect of the COVID-19 pandemic on clinical outcomes in people undergoing neurosurgery, particularly vascular and oncological neurosurgery.

**Method:**

Two investigators independently and systematically searched MEDLINE, EMBASE, the Cochrane Central Register of Controlled Trials (CENTRAL), ClinicalTrail.Gov, and Web of Science to identify relevant studies respecting the criteria for inclusion and exclusion published up to June 30, 2022. The outcomes of our research included mortality rate, length of stay, modified Rankin Score, delay in care, Glasgow outcome scale, and major complications. The risk of bias was assessed using the Methodological Index for Non-randomized Studies (MINORS) checklist.

**Results:**

Two investigators independently and systematically searched 1378 results from MEDLINE, EMBASE, Cochrane database, ClinicalTrail.Gov, and Web of Science and extracted the detailed data from 13 studies that met the review’s eligibility criteria. Two articles reported on patients with intracerebral hemorrhages, five on patients with subarachnoid hemorrhages, four on patients undergoing surgery for neuro-oncology, and in two studies the patients’ conditions were unspecified. A total of 26,831 patients were included in our research. The number who died was significantly increased in the COVID-19 pandemic group (OR 1.52, 95% CI 1.36–1.69, *P* < 0.001). No significant difference was found between the two groups in terms of length of stay (SMD − 0.88, 95% CI − 0.18–0.02, *P* = 0.111), but it differed between regions, according to our subgroup analysis.

**Conclusion:**

Compared to the pre-pandemic group, the number who died was significantly increased in the COVID-19 pandemic group. Meanwhile, the effect of the pandemic on clinical outcomes in people undergoing neurosurgery might differ in different regions, according to our subgroup analysis.

**Supplementary Information:**

The online version contains supplementary material available at 10.1186/s13643-023-02291-5.

## Background

The coronavirus disease-2019 (COVID-19) pandemic has created a global crisis unique in recent history, such as severe economic and social impact, and most importantly, causing severe disruption to the provision of health care around the world [1]. Many countries tried to prevent and control the pandemic by improving personal hygiene, such as wearing masks and washing hands, and avoiding physical contact, such as stopping the service of public transportation and national lockdown [2]. However, these efforts to control the further spread of COVID-19 may have had a significant impact on the diagnosis and treatment processes of diseases in the clinical field [3, 4]. As the medical system was overwhelmed by the global pandemic, other health issues except for COVID-19 received minimal or less attention and most of the non-emergency procedures were deferred [5]. Besides, the pandemic may also have indirect effects on non-COVID patients, on account of the shortage of resources, postponement and cancellation of clinical schedules, and less care for non-urgent and semi-urgent patients. To meet the unusual demand of taking care of COVID-19 patients while simultaneously taking care of those non-COVID patients and reducing exposure risks for vulnerable patients, medical workers had to adapt to physician-led changes [6].

The pandemic also had a profound impact on how neurosurgical patients were clinically managed: a recent global study recognized that since the beginning of the pandemic, neurosurgical operations have more than halved [7]. The pandemic created a reduced capacity to perform surgery and an overall transient but dramatic decrease in surgical volumes at the majority of hospitals, including neurosurgical processes [8, 9]. The GlobalSurg group that published surgical services recently indicated that both elective and emergency were seriously impacted by the COVID-19 pandemic, and in patients infected with COVID-19, there was an increase of up to 23.8% 30-day mortality [10]. A case–control multicenter study described the increased surgical cancellation rates as well as a decline in elective cases, which are responses from surgeons based on responses from surgeons in subjective surveys [11]. The local consequences of the pandemic may have been difficult to manage, due to the growing demands for health care which might have exceeded the capacity of the health system [12–14]. In our research, we evaluated the effect of COVID-19 on clinical outcomes in people undergoing neurosurgery. Cerebrovascular events are being increasingly reported in patients with COVID-19 [15–17]. However, most of the studies have only focused on the phenomenon of increased rates of hemorrhagic stroke in patients who were also positive for COVID-19, and there is little information about the impact of COVID-19 on hemorrhagic stroke patients’ treatment. The CHEST Guideline and Expert Panel Report emphasized the necessity to measure the risk of intracerebral hemorrhage (ICH) in patients who were also positive for COVID-19 because of the increasing anticoagulation requirement [18]. Particular caution is needed for surgery on ICH patients who were combined with COVID-19. A ruptured cerebral aneurysm leading to subarachnoid hemorrhage (aSAH) is known as one of the most severe neurological circumstances with sudden death occurring in 10–15% of these patients, and half of the survivors suffer from permanent disabilities [19]. Due to the pandemic, many problems such as the shortage of ambulances, completing preoperative tests like laboratory tests for SARS-CoV-2, and getting necessary protection equipment may cause a delay in emergency surgery and the golden time to rescue the patient may be missed. Neuro-oncology surgery by endonasal transsphenoidal or craniotomy was resource intensive during the pandemic time, especially with the historical requirement for multi-day hospital admissions, which usually includes an initial recovery period in the intensive care unit (ICU) [20, 21]. The elective surgery or time-limit for surgery for patients who have a brain tumor may have been postponed or even canceled during the pandemic. This unforeseen crisis provided an opportunity and appeal for further streamlining efficient and safe neurosurgical care.

There was no research before trying to investigate the effect of the COVID-19 pandemic on clinical outcomes in people undergoing neurosurgery; thus, we performed this systematic review and meta-analysis to investigate this.

## Method

### Study protocol

We conducted a systematic review and meta-analysis following the Preferred Reporting Items for Systematic Reviews and Meta-Analyses (PRISMA) standards of quality [22]. The study has been registered on the website of INPLASY, with the registration number INPLASY202320025.

### Search strategy

Two investigators independently and systematically searched MEDLINE, EMBASE, the Cochrane Central Register of Controlled Trials (CENTRAL), ClinicalTrail.Gov, and Web of Science to identify relevant studies respecting the criteria for inclusion and exclusion. The search dates were from Dec 2019 up to 30 June 2022. The COVID-19 pandemic was defined as an ongoing global pandemic identified in December 2019. A combination of Medical Subject Headings (MeSH) terms, and keywords (in the title or abstract), was utilized in the research, including *(COVID-19 or SARS-CoV-2 or 2019 Nov) and (neurosurgery or neurosurgical) and (tumor or astrocytoma or ependymoma or glioma or glioblastoma or meningioma or aneurysm or intracranial hemorrhage)*. The search strategy is presented in Supplemental material Table S1. In addition, the reference lists of included studies were screened manually and independently to enhance the search process.

### Eligibility criteria

Studies were included in this systematic review if they met the following criteria of inclusion according to the PEOS framework: (1) patient: any patient with neurosurgical diseases undergoing neurosurgery; (2) exposure: neurosurgery during COVID-19 (exposed population) versus neurosurgery before COVID-19 (non-exposed population); (3) outcomes: including mortality rate, length of stay, modified Rankin Score (mRS), delay in care, Glasgow outcome scale (GOS), and major complications (general complications and surgical complications (e.g., pulmonary embolus, deep vein thrombosis, myocardial infarction, stroke, hematoma, cerebrospinal fluid leak, meningitis…); and (4) study design: case–control studies (more than 10 patients), cross-sectional studies, and retrospective or prospective cohort studies were included.

We excluded those articles that did not meet the inclusion criteria, including (1) RCT, review, commentary, letter, or case reports without a control group. (2) Patients did not receive neurosurgical treatment. (3) Articles were not published in English.

### Study selection and data extraction

We used the EndNote X8 (Thomson Reuters [Scientific] LLC Philadelphia, PA) to manage the retrieved records. Screening and selection of studies were performed independently by two researchers. Data were extracted independently by two researchers respecting the inclusion and exclusion criteria previously mentioned. Specifically, we reviewed articles using titles, abstracts, and full text to collect the following relevant information: author, country of the study, publication year, name of the journal, number of case groups, number of control groups, mortality rate, length of stay, mRS score, delay in care, GOS, and major complications. Disagreements were resolved by discussion or consensus with a third researcher.

### Subgroup analysis

To investigate whether different regions may lead to the different results of the impact of COVID-19 on clinical outcomes in people undergoing neurosurgery, we further performed subgroup analysis according to the different regions, including Europe, Asia, and America.

### Statistical analysis

The STATA software 12.0 (STATA Corp., College Station, Texas, USA) was used to do the statistical analysis. This meta-analysis was conducted using a fixed-effects model. We calculated standardized mean difference (SMD) and 95% confidence interval (CI) for the continuous outcomes. Odds ratio (OR) and 95% CI values were used to evaluate the binary outcomes. A sensitivity analysis was also performed to exclude studies of high risk of bias. Two-tail tests were performed for analyses, and *P* < 0.05 was considered statistical significance.

### Risk of bias

For assessing the risk of bias in the included studies, we used the Methodological Index for Non-randomized Studies (MINORS) checklist [23]. MINORS checklist contains 12 items associated with potential areas of bias. Each item has a score from 0 to 2; thus, overall scores ranged from 0 to 24. The risk of bias was assessed independently by two researchers. In the event of differing opinions between the two researchers, a third researcher was consulted.

## Result

### Search results and study characteristics

**Fig. 1:**
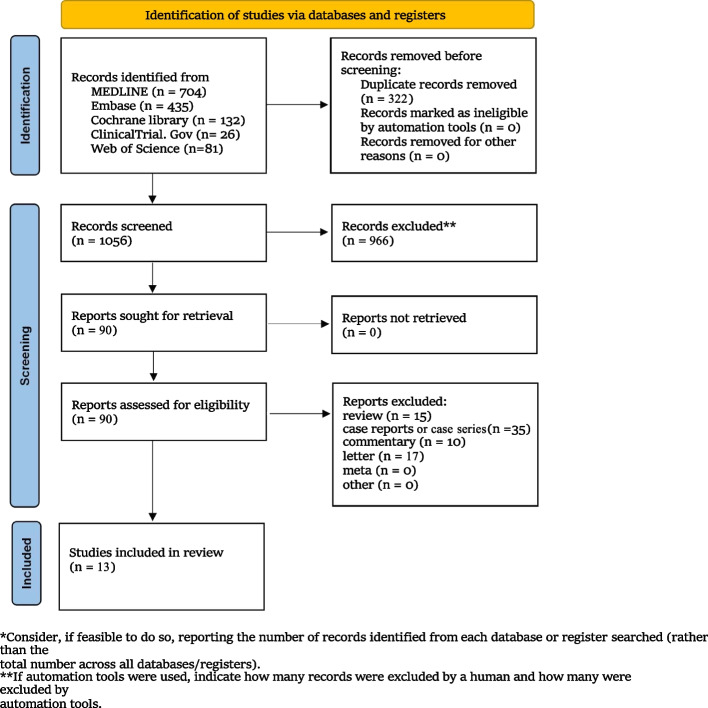
PRISMA flow diagram of study selection

The initial search of the electronic databases yielded 1378 records, of which 322 were duplicates, leaving 1056 records for further screening. After screening based on the title and abstract, we removed 966 studies and 90 records needed for full-text screening. Of these 90, 77 were excluded after reading the full texts, including 35 case reports, 27 letters and comments, 5 systematic reviews and meta-analyses, and 10 reviews. Thus, 13 studies met our eligibility criteria and were included in the review. These included two studies reporting on patients with intracerebral hemorrhages, five on patients with subarachnoid hemorrhages, four studies of patients undergoing surgery for neuro-oncology, and in two studies the patients’ conditions were unspecified. The screening and selection process for studies is shown in Fig. 1. The summary of the characteristics of the included articles is presented in Table 1.

**Table 1:** Basic information of studies

Authors & Year	Study Design	Country	Disease	Number of cases	Number of controls	Outcomes
Amarouche 2021 [24]	Retrospective	United Kingdom	Transsphenoidal Pituitary Surgery	27	39	Length of stay Delay in care
Amoo 2021 [25]	Retrospective	Ireland	Neuro-oncology	127	139	Mortality Length of Stay
Ashkan 2021 [26]	Prospective	United Kingdom	Unspecific neurosurgery	206	453	Mortality
Bajunaid 2020 [11]	Retrospective	Kingdom of Saudi Arabia	Unspecific neurosurgery	305	545	Mortality Length of Stay Delay in Care Major Complications
Fiorindi 2022 [27]	Retrospective	Italy	Aneurismal Subarachnoid Hemorrhage	72	179	Mortality Length of Stay Delay in Care Glasgow Outcome Scale
Han 2021 [2]	Retrospective	Korea	Intracerebral Hemorrhage	83	255	mRS Score Delay in care
Kashefiolasl 2022 [28]	Retrospective	Germany	Aneurysmal Subarachnoid Hemorrhage	56	84	mRS Score Delay in Care
Mallari 2021 [29]	Retrospective	United States of America	Neuro-oncology	132	163	Mortality Length of Stay Major Complications
Miękisiak 2022 [30]	NA	Poland	Intracranial Aneurysms	3399	18,402	Mortality
Norman 2021 [6]	Retrospective	United States of America	Neuro-oncology	112	166	Mortality Delay in Care
Qureshi 2021^a^ [31]	NA	United States of America	Subarachnoid Hemorrhage	86	376	Mortality Length of Stay
Qureshi 2022^a^ [32]	NA	United States of America	Intracerebral Hemorrhage	154	667	Mortality Length of Stay
Theofanopoulos 2021 [33]	Retrospective	Greece	Aneurysmal Subarachnoid Hemorrhage	31	68	Mortality Length of Stay

^a^Studies compare COVID-19 patients with non COVID-19 patients

### Risk of bias

Based on the MINORS quality checklist, the quality of 13 included studies was considered acceptable, with an average score of 16.7, and none of them was excluded (Supplementary Table S2). However, sensitivity analysis indicated that one article [30] demonstrated publication bias in the result of mortality (Figure S1). The result that excludes the article is demonstrated in Figure S2. And another article [11]. demonstrated publication bias in the result of the length of stay (Figure S3). The result that excludes this article was demonstrated in Figure S4.

### Mortality rate

Due to the lack of available data, we only conducted a meta-analysis of mortality, length of stay, and subgroup analysis according to the different regions.

**Fig. 2:**
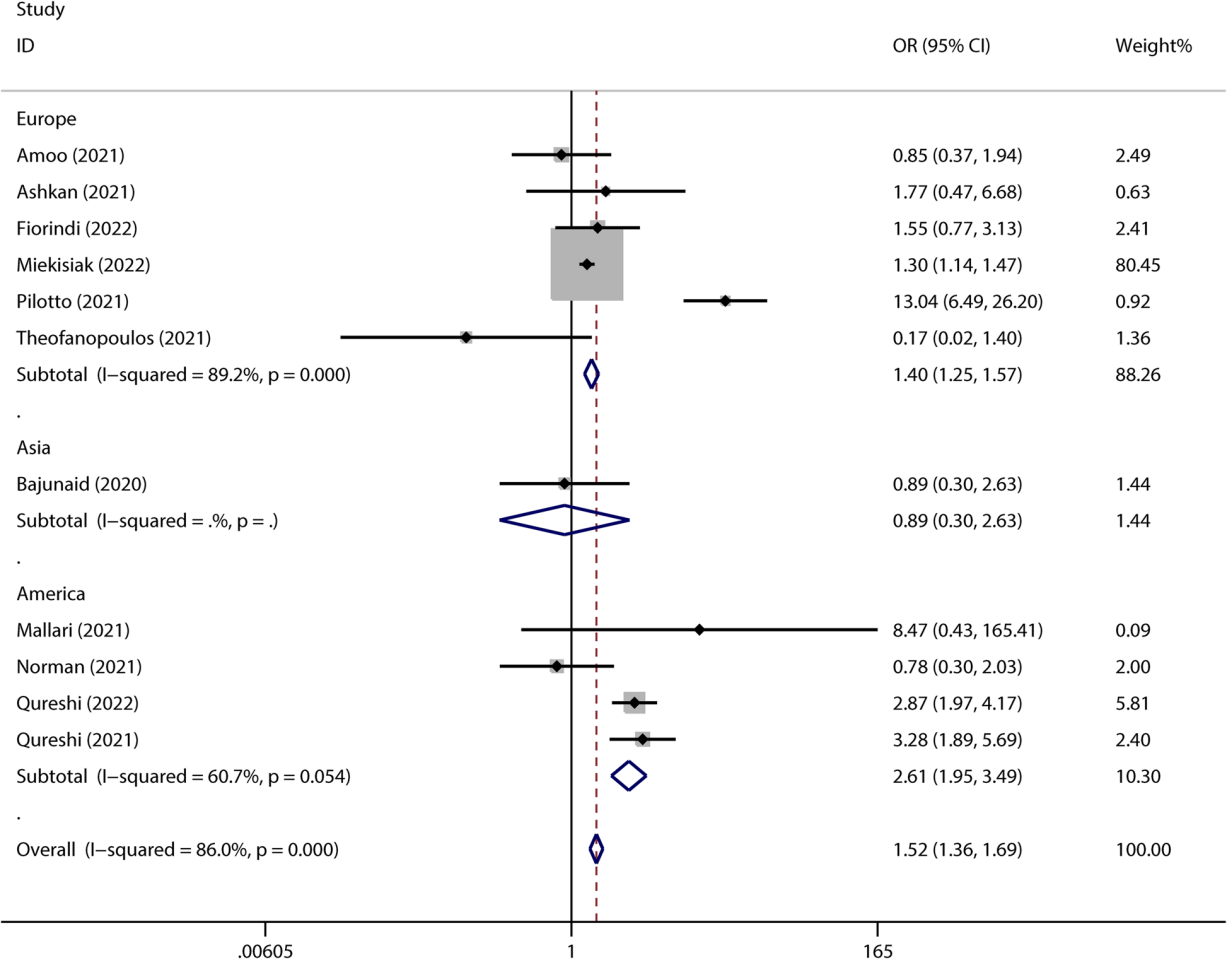
The forest plot of the mortality rate

Eleven studies reported the mortality rate. There was a significant difference between the two groups in terms of mortality (OR 1.52, 95% CI 1.36–1.69, *P* < 0.001; Fig. 2). In terms of mortality, there was a significant difference in the European group (OR 1.40, 95% CI 1.25–1.57, *P* < 0.001; Fig. 2) and American group (OR 2.61, 95% CI 1.95–3.49, *P* < 0.001; Fig. 2). However, no significant difference was found in the Asian group (OR 0.89, 95% CI 0.30–2.63, *P* = 0.836; Fig. 2).

### Length of stay

**Fig. 3:**
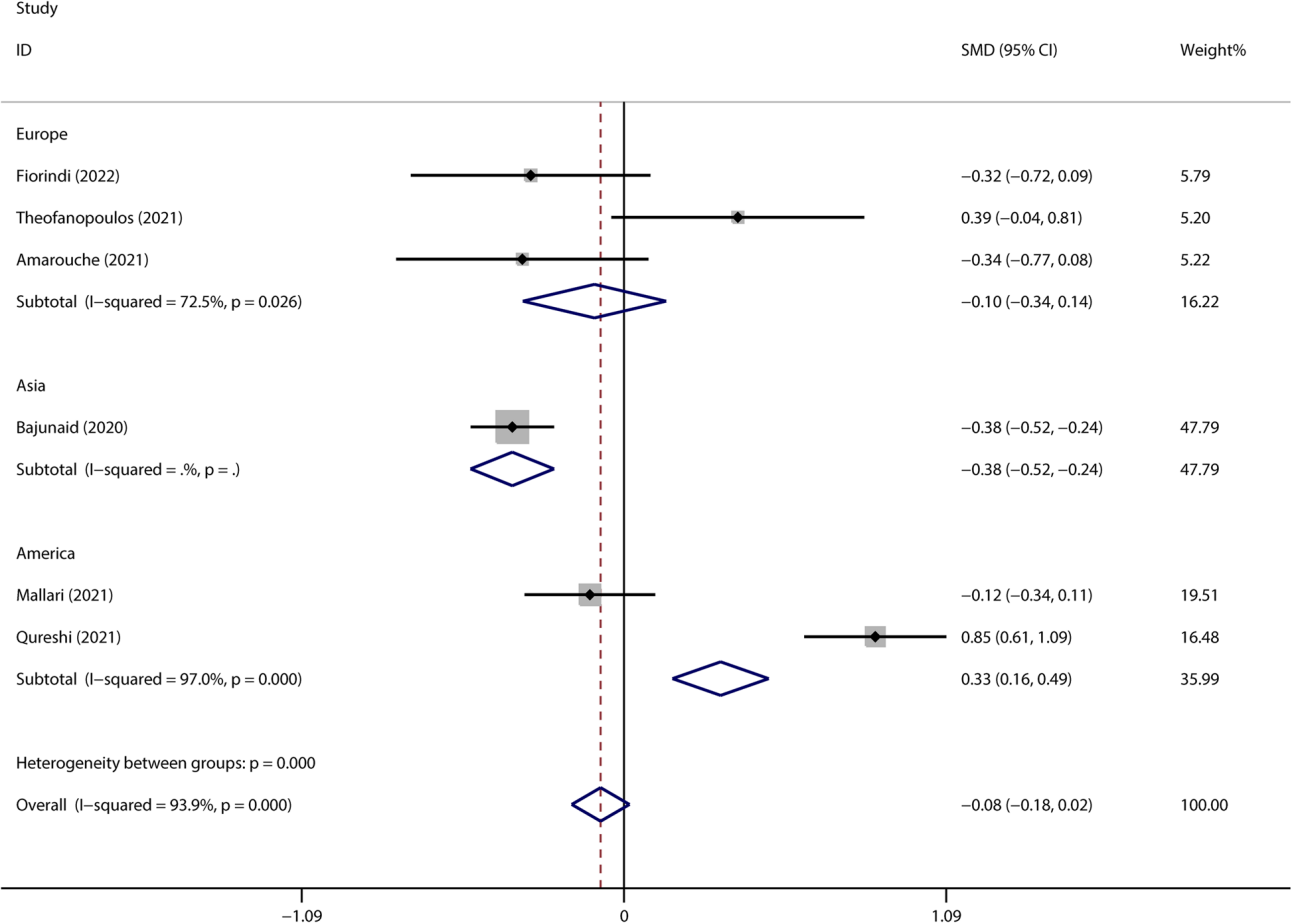
The forest plot of the length of stay

Six studies reported the length of stay. No significant difference was found between the two groups in terms of length of stay (SMD − 0.88, 95% CI − 0.18–0.02, *P* = 0.111; Fig. 3). The length of stay was significantly prolonged in the American group (SMD 0.33, 95% CI 0.16–0.49, *P* < 0.001; Fig. 3) but was significantly shortened in the Asian group (SMD − 0.38, 95% CI − 0.52–0.24, *P* < 0.001; Fig. 3). However, no significant difference was found in the European group (OR − 0.10, 95% CI − 0.34–0.14, *P* = 0.418; Fig. 3).

### mRS score

Two studies reported the mRS score. In the study by Han et al. [2], clinical outcomes of patients in the COVID-19 group were worse at 3-month follow-up (mRS ≤ 0–2; 33.7% versus 46.7%; *p* = 0.039). In the study by Kashefiolasl et al. [28], two groups had similar rates of favorable outcomes (32% versus 37%).

### Delay in care

Five studies reported the delay in care. In the study by Amarouche et al. [24], the median duration of anesthesia was 43 min during the pandemic and 25 min pre-pandemic. In the study by Fiorindi et al. [27], the diagnostic delay increased significantly (+ 68%) in the COVID-19 group versus pre-pandemic (1.06 versus 0.63 days, *p* = 0.030), while therapeutic delay did not significantly differ between the two groups (0.89 versus 0.74 days, *p* = 0.183). In the study by Han et al. [2], symptom onset or detection-to-door time (56.0 min versus 40.0 min; *P* = 0.016) and median door-to-intensive treatment time significantly differed between the COVID-19 and pre-COVID-19 groups [349.0 min versus 184.0 min, *P* < 0.001]. In the study by Kashefiolasl et al. [28], the delay in hospital admission days between the groups is not significantly different (0.94 ± 1.45 versus 0.77 ± 1.3). In the study by Norman et al. [6], there were significantly more delays in care during the pandemic (8.0% versus 1.8%, *P* = 0.016).

### Glasgow Outcome Scale

Two studies reported the Glasgow Outcome Scale. In the study by Fiorindi et al. [27], patients with poor outcomes rate (GOS at discharge 1–3) were higher during the COVID-19 pandemic compared to the non-COVID period (54.2% versus 40.2%, *p* = 0.044). In the study by Han et al. [2], no significant difference was found between the two groups in terms of initial GCS (11.6 ± 4.00 versus 10.9 ± 4.30, *P* = 0.175).

### Major complications

Three studies reported the major complications. In the study by Amarouche et al. [24], no COVID-19-related complications were seen. In the study by Bajunaid et al. [24], two separate groups of complications were reported: general and craniospinal. General complications occurred in 3.61% of patients during COVID-19 compared with 5.7% pre-COVID-19, whereas craniospinal complications occurred in 9.18% of patients during COVID-19 compared with 9.17% pre-COVID-19. In the study by Mallari et al. [29], no significant differences were found comparing pandemic and pre-pandemic cohorts in overall complication rates (11 versus 20, *P* = 0.81).

## Discussion

### Intracerebral hemorrhage

ICH is considered to be the most disastrous stroke subtype with a mortality rate of over 40% and a significantly high morbidity rate among survivors [34]. About 40% of deaths occur within the first month after ICH, and only 20% of patients will fully recover approximately [35, 36]. Motor, memory, and language deficits, as severe morbidities, may be caused by hemorrhage-related injury [34]. Increasing evidence has concluded that patients with COVID-19 could present neurological symptoms, while cerebrovascular diseases are one of the most common comorbidities [37, 38]. The rate of intracranial hemorrhage associated with COVID-19 was much higher than other respiratory viruses, for instance, the influenza virus (OR 2.85, 1.35–6.02) [39]. It is unclear whether the manifestations of cerebrovascular disease are caused by a direct viral infection—a mechanism suggested by olfactory nerve leading to the retrograde brain infection—or an indirect action mediated by inflammatory hyperactivation, known as the cytokine storm, resulting in severe immune and coagulation systems dysfunction, bringing about elevated D-dimer levels and intravascular disseminated intravascular coagulation (DIC) [40]. In a previous review and meta-analysis, You et al. identified alcohol intake and hypertension as important risk factors for the significant increase of ICH during the pandemic [41]. Although the admissions of hemorrhagic stroke during the pandemic period are less than during the pre-pandemic period, the proportion of hemorrhagic stroke hospitalizations in all types of stroke is significantly increased.

The results of You et al. [42]. also indicated that the admission number of ICH was 618 in the COVID-19 pandemic group and 461 in the pre-pandemic group, with a corresponding rate raised. In addition, higher levels of D-dimer and tissue plasminogen activator (tPA) plasma were reported in patients with severe COVID-19, both of which are related to an increased propensity for hemorrhagic complications [43]. It is notable to indicate that in the case series of Abbas et al., 77% of the COVID-19 patients had poor outcomes (mRS 3–6). The mortality rate was 59% [44]. In the literature of Berikol et al. [45], they evaluated the duration of the waiting time and found that 60.3% of the ICH patients waited over 6 h, 35.9% waited for 6 to 24 h, and 3.8% waited more than 1 day in the emergency department. The large number of patients admitted to the emergency room, insufficient intensive care and service beds, and the extension of referral times due to the density and occupancy of other medical centers can all contribute to increased emergency department wait times. Lawton et al. [46] identified patients hospitalized with ICH and COVID-19 infection compared with the control group of COVID-19-negative ICH patients has demonstrated that these patients were younger, had a worse prognosis, and longer lengths of hospital stay. According to the multivariable analysis, an unfavorable 90-day functional outcome was associated with worse medical intensive care, low GCS, and advanced age [2]. The time from the onset of COVID-19 symptoms to the diagnosis of the development of ICH varies. Median times of 1 or 1.5 days have been observed in some studies [47] and up to 32 in others [48]. Part of this variability may be due to the deep sedation of ICU patients masking the typical symptoms of intracerebral hemorrhage. Clinicians should strongly suspect the possibility of ICH in critically ill patients with COVID-19 infection and receive the treatment of anticoagulants and admit to ICU [49].

### Subarachnoid hemorrhage

SAH is a serious medical emergency characterized by the existence of blood in the subarachnoid area, cerebral parenchyma, and occasional intraventricular [50]. Non-traumatic SAH is usually caused by a ruptured aneurysm, called aSAH [51]. Acute SAH due to aneurysmal rupture is a severe vascular disease leading to approximately 5% of strokes and causes an extremely large burden of mortality and morbidity [52]. One third of patients died before arriving at the hospital or within the first days after aneurysmal rupture, one third of patients are confronted with long-term deficits and complications of neurological function, and the last third of patients recover to normal life [53, 54]. The most common risk factor for aSAH is hypertension [54]. Moreover, Qin et al. [55]. demonstrated that COVID-19 can induce cytokine storm, named hypercytokinemia, causing elevated systemic inflammation accompanied by high levels of mediators, including TNFa, IL-1b, and IL-6. The consequence of this progress is vascular injury [56]. The SARS-CoV-2 virus has been reported to attach surface angiotensin-converting enzyme 2 (ACE-2) receptor to enter cells, thus causing endothelial injury as well. Endothelial injury may be caused by direct or indirect endothelial toxicity which may explain the deformation of arterial wall and the development or rupture of aneurysm in COVID-19 patients [57]. The systemic infection of the COVID-19 virus may play an important role in the development of cerebral aneurysms and secondary aSAH. In the patients analyzed in the study by Akbik et al. [58], they observed remarkable elevation of serum inflammatory markers, such as CRP (ranged from 27 to 6) and D-dimers (ranged from 1134 to 4000).

In a retrospective cohort study [59], they reported that aSAH in the COVID-19 era may be related to delayed manifestation and consequent increase in brain vasospasm, delayed cerebral ischemia, re-rupture of aneurysmal, higher in-hospital mortality rate, and more terminal care disposition. Moreover, according to the study of Theofanopoulos et al., when compared to the pre-COVID-19 period (previous year to the first COVID-19 infection case in 2020), there was a noteworthy increase of 1.5 times in the number of patients admitted to their hospital with spontaneous SAH during the COVID-19 pandemic (from the first COVID-19 infection case to the lifting of the blockade in Greece) [33]. By contrast, studies from St. Michael’s Hospital, Toronto, Canada [60], and another by Lariboisière Hospital in Paris, France [61], both reported a significant reduction of SAH admissions during the beginning of the pandemic. During the beginning of the pandemic, the in-hospital pathways were heavily affected, and many regions were even blocked.

### Neuro-oncology

During the COVID-19 pandemic, multiple elective neurosurgeries were canceled due to the shortage of ICU capacity, the transformation of regular postoperative wards into COVID-19 units, the requirement for the preservation of personal protective equipment (PPE), the closing of neurorehabilitation, and most vitally the protective measures taken to avoid the newly postoperative patients from getting COVID-19 infection [62, 63]. However, a small group of neurosurgical procedures cannot be dissolved, especially those that have been deemed necessary to operate on to avert impending death or irreversible disabilities to the patient. This is especially necessary the tumor cases located in the brain or spine in our practice of neurosurgical oncology. Operation is essential to reduce the massive effect of the tumor on the brain or spinal cord to preserve or improve neurological function [62].

Glioblastoma (GBM) is the most common primary malignant brain tumor, accounting for nearly 48% of all primary central nervous system malignant tumors and 57% of all gliomas [64]. GBM patients are supposed to be one of the most vulnerable patient populations during the COVID-19 pandemic, mainly on account of the increasing incidence of GBM in elderly patients, treatment-related immunosuppression, and requirement for the frequent visits to the hospital. The mortality rate in patients with primary brain tumors was similar to that in patients with nonthoracic solid tumors [65, 66]. According to Amoo, during the period of the COVID-19 pandemic in 2020, gliomas accounted for 47.24% (*n* = 60) of treated neuro-oncological tumors, while 85% of the gliomas (*n* = 51) were high-grade glial tumors (WHO 3 or 4). During the same period in 2019, 40.3% (*n* = 56) of neuro-oncological patients had glioma, while 78.6% (*n* = 44) of them were high-grade. In the year 2020, 16.7% (*n* = 10) of patients undergoing glioma surgery were electively admitted from home, fewer than the patients in 2019 (30.4%, *n* = 17) [25]. In the study by Mallari et al. [29], two cohorts of brain tumor patients, which are well-matched in age, preoperative ASA physical status class, operation type, and tumor pathology, after carrying out a streamlined care protocol, the utilization of ICU of surgeries decreased from 54 to 29% and hospital length of stay of less than 1 day increased from 12 to 41%. The delay in care and mortality rate have no statistical significance in our research. We assumed that elective surgery may not be strongly affected by the short-term delay in care. In addition, some patients who need emergency surgery may not have been able to arrive at the hospital in time and passed away at home or on the way. So we suggest that our data may show some survivor deviation. Moreover, the sample size of our research is not large enough to answer the question.

Meningioma, which originates from the leptomeninges, is classically a benign neoplasm [67]. Although post-pandemic neuro-oncology is increasing in complexity and severity compared to pre-pandemic levels, the surgical treatment for meningioma is not affected. Therefore, Zou et al. [68] suggested that under the circumstances of limited medical resources, meningioma patients can be postponed surgery and initially undergo conservative treatment.

Pituitary adenomas, accounting for 10–25% of all intracranial tumors, are usually benign. Gu et al. evaluated the differential expression level of ACE2 between pituitary adenoma tissues and normal pituitary glands to identify whether the pituitary gland can be affected by SARS-CoV-2 as a target organ. The results suggested that in pituitary glands and pituitary adenoma, ACE2 has a low expression level at the protein and mRNA levels. Low ACE2 might reduce the SARS-CoV-2 entry or local viral load. It may also deteriorate the stress resistance function of the organ and fail to act as a protective role. Next, they compared the profiles of pituitary hormone between the cohort of COVID-19 patients and healthy controls in this study, which is well matched in age and gender, and found significantly increased levels of serum PRL and ACTH in the COVID-19 group [69].

Besides, it is noteworthy that the mental health of the patients who undergo the operation treatment is especially concerning. Because of the first wave of the COVID-19 pandemic in early 2020, both patients and their relatives exhibited significantly higher levels of depression, distress, and anxiety. Quality of life between patients and their families is correlated, informing the need to focus on the entire family for mental health interventions during the pandemic [70].

### Meta-analysis

Compared to the pre-pandemic group, the number who died was significantly increased in the COVID-19 pandemic group. At the beginning of the wave, it was obvious that the healthcare system had not prepared for such a pandemic of this magnitude; thus, the exact number of lives lost was countless. The increased mortality rate may be due to overrun ICUs and insufficient ventilators stay-at-home that avert a collapse of the medical system. Meanwhile, the infection of SARS-CoV-2 is crucial to the death of patients who underwent neurosurgery. The GlobalSurg reported a 23.8% of 30-day perioperative mortality rate in COVID-19 patients who underwent elective or emergency surgery [71]. And the complications caused by COVID-19 can affect a wide range of organ systems. It was suggested that a hypercoagulable and inflammatory state caused by COVID-19 could result in intracranial hemorrhage or acute ischemic stroke even in patients without apparent risk factors found [72]. No significant difference was found between the two groups in terms of length of stay. The length of stay was significantly prolonged in the American group but was significantly shortened in the Asian group, and no significant difference was found in the European group. The length of stay may depend on the severity of the pandemic, the development of the economic and healthcare system, and the availability of vaccination against COVID-19 in the region.

### Limitation

There are some limitations of our study. First, a few studies were included in the review. Then, because of the heterogeneity and insufficient data of these studies, we were unable to conduct a meta-analysis on all of our pre-specified outcomes. In addition, the effects of the pandemic on outcomes might differ in different phases of the pandemic, availability of vaccination of COVID-19, or variant types of SARS-CoV-2, and further sub-group analyses were not possible.

## Conclusion

Our studies suggested that the COVID-19 pandemic caused a negative effect on the outcomes of neurosurgery. Compared to the pre-pandemic group, the number who died was significantly increased in the COVID-19 pandemic group. Meanwhile, the effect of a pandemic on clinical outcomes in people undergoing neurosurgery might differ in different regions, according to our subgroup analysis.

### Supplementary Information

**Additional file 1: Table S1.** Search strategies and results.** Table S2.** Risk of bias based on MINORS quality assessment. **Figure S1.** Sensitivity analysis of the number of mortality. **Figure S2.** Forest plots for the number of mortality after excluding the article of Miękisiak et.al. **Figure S3.** sensitivity analysis of the length of hospital stay. **Figure S4.** Forest plots for the length of stay after excluding the article of Bajunaid et al.

## Data Availability

Not applicable.

## Abbreviations

COVID-19: Coronavirus disease-2019
CENTRAL: Cochrane Central Register of Controlled Trials
aSAH: Subarachnoid hemorrhage
ICU: Intensive care unit
PRISMA: Preferred Reporting Items for Systematic Reviews and Meta-Analyses
MeSH: Medical Subject Headings
DIC: Disseminated intravascular coagulation
tPA: Tissue plasminogen activator
ACE-2: Angiotensin-converting enzyme 2
PPE: Personal protective equipment
GBM: Glioblastoma

## References

[1] Ceraudo M, Balestrino A, Cama A, Macrina G, Piatelli G, Consales A (2021). Pediatric neurosurgery after the COVID-19 pandemic: management strategies from a single pediatric hospital in Italy. World Neurosurg.

[2] Han HJ, Park KY, Kim J, Lee W, Lee YH, Jang CK, Cho KC, Park SK, Chung J, Kwon YS (2021). Delays in Intracerebral hemorrhage management is associated with hematoma expansion and worse outcomes: changes in COVID-19 era. Yonsei Med J.

[3] Durbin RP (1975). Letter: Acid secretion by gastric mucous membrane. Am J Physiol.

[4] Weiner HL, Adelson PD, Brockmeyer DL, Maher CO, Gupta N, Smyth MD, Jea A, Blount JP, Riva-Cambrin J, Lam SK, et al. Editorial. Pediatric neurosurgery along with children's hospitals' innovations are rapid and uniform in response to the COVID-19 pandemic. J Neurosurg Pediatr. 2020;26(1):3–5. 10.3171/2020.4.PEDS20240. 10.3171/2020.4.PEDS20240 PMC7164397 32302988

[5] Collaborative C (2020). Elective surgery cancellations due to the COVID-19 pandemic: global predictive modelling to inform surgical recovery plans. Br J Surg.

[6] Norman S, Ramos A, Giantini Larsen AM, Bander E, Goldberg J, Parker W, Juthani RG (2021). Impact of the COVID-19 pandemic on neuro-oncology outcomes. J Neurooncol.

[7] Jean WC, Ironside NT, Sack KD, Felbaum DR, Syed HR (2020). The impact of COVID-19 on neurosurgeons and the strategy for triaging non-emergent operations: a global neurosurgery study. Acta Neurochir (Wien).

[8] Khalafallah AM, Jimenez AE, Lee RP, Weingart JD, Theodore N, Cohen AR, Tamargo RJ, Huang J, Brem H, Mukherjee D (2020). Impact of COVID-19 on an Academic Neurosurgery Department: The Johns Hopkins experience. World Neurosurg.

[9] Fontanella MM, De Maria L, Zanin L, Saraceno G, Terzi di Bergamo L, Servadei F, Chaurasia B, Olivi A, Vajkoczy P, Schaller K (2020). Neurosurgical practice during the severe acute respiratory syndrome coronavirus 2 (SARS-CoV-2) pandemic: a worldwide survey. World Neurosurg.

[10] Collaborative CO (2020). Mortality and pulmonary complications in patients undergoing surgery with perioperative SARS-CoV-2 infection: an international cohort study. Lancet.

[11] Bajunaid K, Alqurashi A, Alatar A, Alkutbi M, Alzahrani AH, Sabbagh AJ, Alobaid A, Barnawi A, Alferayan AA, Alkhani AM (2020). Neurosurgical procedures and safety during the COVID-19 pandemic: a case-control multicenter study. World Neurosurgery.

[12] Toner E, Waldhorn R, Maldin B, Borio L, Nuzzo JB, Lam C, Franco C, Henderson DA, Inglesby TV, O'Toole T (2006). Hospital preparedness for pandemic influenza. Biosecur Bioterror.

[13] Zoutman DE, Ford BD, Melinyshyn M, Schwartz B (2010). The pandemic influenza planning process in Ontario acute care hospitals. Am J Infect Control.

[14] Reidy M, Ryan F, Hogan D, Lacey S, Buckley C (2015). Preparedness of hospitals in the Republic of Ireland for an influenza pandemic, an infection control perspective. BMC Public Health.

[15] Klok FA, Kruip M, van der Meer NJM, Arbous MS, Gommers D, Kant KM, Kaptein FHJ, van Paassen J, Stals MAM, Huisman MV (2020). Confirmation of the high cumulative incidence of thrombotic complications in critically ill ICU patients with COVID-19: an updated analysis. Thromb Res.

[16] Mao L, Jin H, Wang M, Hu Y, Chen S, He Q, Chang J, Hong C, Zhou Y, Wang D (2020). Neurologic manifestations of hospitalized patients with coronavirus disease 2019 in Wuhan China. JAMA Neurol.

[17] Markus HS, Brainin M (2020). COVID-19 and stroke-A global World Stroke Organization perspective. Int J Stroke.

[18] Moores LK, Tritschler T, Brosnahan S, Carrier M, Collen JF, Doerschug K, Holley AB, Jimenez D, Le Gal G, Rali P (2020). Prevention, diagnosis, and treatment of VTE in patients with coronavirus disease 2019: CHEST Guideline and Expert Panel Report. Chest.

[19] Nguyen TN, Abdalkader M, Jovin TG, Nogueira RG, Jadhav AP, Haussen DC, Hassan AE, Novakovic R, Sheth SA, Ortega-Gutierrez S (2020). Mechanical thrombectomy in the era of the COVID-19 pandemic: emergency preparedness for neuroscience teams: a guidance statement from the society of vascular and interventional neurology. Stroke.

[20] Dasenbrock HH, Liu KX, Devine CA, Chavakula V, Smith TR, Gormley WB, Dunn IF (2015). Length of hospital stay after craniotomy for tumor: a National Surgical Quality Improvement Program analysis. Neurosurg Focus.

[21] Senders JT, Muskens IS, Cote DJ, Goldhaber NH, Dawood HY, Gormley WB, Broekman MLD, Smith TR (2018). Thirty-day outcomes after craniotomy for primary malignant brain tumors: a national surgical quality improvement program analysis. Neurosurgery.

[22] Page MJ, McKenzie JE, Bossuyt PM, Boutron I, Hoffmann TC, Mulrow CD, Shamseer L, Tetzlaff JM, Akl EA, Brennan SE (2021). The PRISMA 2020 statement: an updated guideline for reporting systematic reviews. Bmj.

[23] Slim K, Nini E, Forestier D, Kwiatkowski F, Panis Y, Chipponi J (2003). Methodological index for non-randomized studies (minors): development and validation of a new instrument. ANZ J Surg.

[24] Amarouche M, Borg A, Halliday J, Cudlip SA. Safety of transsphenoidal pituitary surgery during the COVID-19 pandemic and comparison to the prepandemic era. J Neurol Surg Part B: Skull Base. 2021;82(SUPPL 2). 10.1055/s-0041-1725446. 10.1055/s-0041-1730352 PMC9272276 35833005

[25] Amoo M, Horan J, Gilmartin B, Nolan D, Corr P, MacNally S, Husien MB, Javadpour M (2021). The provision of neuro-oncology and glioma neurosurgery during the SARS-CoV-2 pandemic: a single national tertiary centre experience. Ir J Med Sci.

[26] Ashkan K, Jung J, Velicu AM, Raslan A, Faruque M, Kulkarni P, Bleil C, Hasegawa H, Kailaya-Vasan A, Maratos E (2021). Neurosurgery and coronavirus: impact and challenges—lessons learnt from the first wave of a global pandemic. Acta Neurochir.

[27] Fiorindi A, Vezzoli M, Doglietto F, Zanin L, Saraceno G, Agosti E, Barbieri A, Bellocchi S, Bernucci C, Bongetta D (2022). Aneurismal subarachnoid hemorrhage during the COVID-19 outbreak in a Hub and Spoke system: observational multicenter cohort study in Lombardy Italy. Acta Neurochir (Wien).

[28] Kashefiolasl S, Qasem LE, Brawanski N, Funke M, Keil F, Hattingen E, Foerch C, Seifert V, Prinz VM, Czabanka M, et al. Impact of COVID-19 pandemic on treatment management and clinical outcome of aneurysmal subarachnoid hemorrhage - a single-center experience. Front Neurol. 2022;13:836422. 10.3389/fneur.2022.836422. 10.3389/fneur.2022.836422 PMC8978712 35386414

[29] Mallari RJ, Avery MB, Corlin A, Eisenberg A, Hammond TC, Martin NA, Barkhoudarian G, Kelly DF (2021). Streamlining brain tumor surgery care during the COVID-19 pandemic: a case-control study. PLoS One.

[30] Miękisiak G, Fercho J, Pettersson SD, Szmuda T, Słoniewski P (2022). Impact of COVID-19 on incidence and treatment of intracranial aneurysms in Poland: a national study. Neurol Neurochir Pol.

[31] Qureshi AI, Baskett WI, Huang W, Shyu D, Myers D, Lobanova I, Ishfaq MF, Naqvi SH, French BR, Siddiq F (2021). Subarachnoid hemorrhage and COVID-19: an analysis of 282,718 patients. World Neurosurg.

[32] Qureshi AI, Baskett WI, Huang W, Myers D, Lobanova I, Ishfaq MF, Naqvi SH, French BR, Chandrasekaran PN, Siddiq F (2022). Intracerebral hemorrhage and coronavirus disease 2019 in a cohort of 282,718 hospitalized patients. Neurocrit Care.

[33] Theofanopoulos A, Fermeli D, Boulieris S, Kalantzis G, Kefalopoulou Z, Panagiotopoulos V, Papadakos D, Constantoyannis C (2021). Effects of COVID-19 on the admissions of aneurysmal subarachnoid hemorrhage: the West Greece experience. Neurol Sci.

[34] Rymer MM (2011). Hemorrhagic stroke: intracerebral hemorrhage. Mo Med.

[35] Atillasoy J, Leasure AC, Sheth KN (2021). Intracerebral hemorrhage in COVID-19 infection. World Neurosurg.

[36] Karimy JK, Reeves BC, Kahle KT (2020). Targeting TLR4-dependent inflammation in post-hemorrhagic brain injury. Expert Opin Ther Targets.

[37] Tsivgoulis G, Palaiodimou L, Zand R, Lioutas VA, Krogias C, Katsanos AH, Shoamanesh A, Sharma VK, Shahjouei S, Baracchini C (2020). COVID-19 and cerebrovascular diseases: a comprehensive overview. Ther Adv Neurol Disord.

[38] Wang Z, Yang Y, Liang X, Gao B, Liu M, Li W, Chen Z, Wang Z. COVID-19 associated ischemic stroke and hemorrhagic stroke: incidence, potential pathological mechanism, and management. Front Neurol. 2020;11:571996. 10.3389/fneur.2020.571996. 10.3389/fneur.2020.571996 PMC7652923 33193019

[39] Daly SR, Nguyen AV, Zhang Y, Feng D, Huang JH (2021). The relationship between COVID-19 infection and intracranial hemorrhage: a systematic review. Brain Hemorrhages.

[40] Fraiman P, Godeiro Junior C, Moro E, Cavallieri F, Zedde M. COVID-19 and cerebrovascular diseases: a systematic review and perspectives for stroke management. Front Neurol. 2020;11:574694. 10.3389/fneur.2020.574694. 10.3389/fneur.2020.574694 PMC7674955 33250845

[41] You Y, Niu Y, Sun F, Zhang J, Huang S, Ding P, Wang X (2021). Impact of COVID-19 pandemic on haemorrhagic stroke admissions: a systematic review and meta-analysis. BMJ Open.

[42] You Y, Niu Y, Sun F, Zhang J, Huang S, Ding P, Wang X. Impact of COVID-19 pandemic on haemorrhagic stroke admissions: a systematic review and meta-analysis. BMJ Open. 2021;11(12). 10.1136/bmjopen-2021-050559. 10.1136/bmjopen-2021-050559 PMC8671851 34907050

[43] Becker RC (2020). COVID-19 update: Covid-19-associated coagulopathy. J Thromb Thrombolysis.

[44] Abbas R, El Naamani K, Sweid A, Schaefer JW, Bekelis K, Sourour N, Elhorany M, Pandey AS, Tjoumakaris S, Gooch MR (2021). Intracranial hemorrhage in patients with coronavirus disease 2019 (COVID-19): a case series. World Neurosurg.

[45] Berikol G, Berikol GB, Dogan H (2021). Retrospective analysis of intracranial hemorrhages in the COVID-19 pandemic. Turk Neurosurg.

[46] Lawton MT, Alimohammadi E, Bagheri SR, Bostani A, Vaziri S, Karbasforoushan A, Mozaffari K, Bukani MB, Abdi A (2021). Coronavirus disease 2019 (COVID-19) can predispose young to Intracerebral hemorrhage: a retrospective observational study. BMC Neurol.

[47] Shahjouei S, Naderi S, Li J, Khan A, Chaudhary D, Farahmand G, Male S, Griessenauer C, Sabra M, Mondello S, et al. Risk of stroke in hospitalized SARS-CoV-2 infected patients: a multinational study. EBioMedicine. 2020;59:102939. 10.1016/j.ebiom.2020.102939. 10.1016/j.ebiom.2020.102939 PMC7429203 32818804

[48] Benger M, Williams O, Siddiqui J, Sztriha L (2020). Intracerebral haemorrhage and COVID-19: clinical characteristics from a case series. Brain Behav Immun.

[49] Margos NP, Meintanopoulos AS, Filioglou D, Ellul J (2021). Intracerebral hemorrhage in COVID-19: a narrative review. J Clin Neurosci.

[50] Macdonald RL (2014). Delayed neurological deterioration after subarachnoid haemorrhage. Nat Rev Neurol.

[51] Alotaibi AS, Mahroos RA, Al Yateem SS, Menezes RG (2022). Central nervous system causes of sudden unexpected death: a comprehensive review. Cureus.

[52] Grasso G, Alafaci C, Macdonald RL (2017). Management of aneurysmal subarachnoid hemorrhage: state of the art and future perspectives. Surg Neurol Int.

[53] Etminan N, Chang HS, Hackenberg K, de Rooij NK, Vergouwen MDI, Rinkel GJE, Algra A (2019). Worldwide incidence of aneurysmal subarachnoid hemorrhage according to region, time period, blood pressure, and smoking prevalence in the population: a systematic review and meta-analysis. JAMA Neurol.

[54] Kundra S, Mahendru V, Gupta V, Choudhary AK (2014). Principles of neuroanesthesia in aneurysmal subarachnoid hemorrhage. J Anaesthesiol Clin Pharmacol.

[55] Qin C, Zhou L, Hu Z, Zhang S, Yang S, Tao Y, Xie C, Ma K, Shang K, Wang W (2020). Dysregulation of immune response in patients with coronavirus 2019 (COVID-19) in Wuhan China. Clin Infect Dis.

[56] Mehta P, McAuley DF, Brown M, Sanchez E, Tattersall RS, Manson JJ, Collaboration HAS, UK,  (2020). COVID-19: consider cytokine storm syndromes and immunosuppression. Lancet.

[57] Wang D, Hu B, Hu C, Zhu F, Liu X, Zhang J, Wang B, Xiang H, Cheng Z, Xiong Y (2020). Clinical characteristics of 138 hospitalized patients with 2019 novel coronavirus-infected pneumonia in Wuhan China. JAMA.

[58] Cezar-Junior AB, Faquini IV, Silva JLJ, de Carvalho Junior EV, Lemos L, Freire Filho JBM, de Lira Filho HT, Pontes ECA, Almeida NS, Azevedo-Filho HRC (2020). Subarachnoid hemorrhage and COVID-19: association or coincidence?. Medicine (Baltimore).

[59] Akbik F, Yang C, Howard B, Grossberg J, Danyluk L, Martin K, Alawieh A, Rindler R, Tong F, Barrow D (2021). Delayed presentations, increased complications, and worse outcomes after aneurysmal subarachnoid hemorrhage in the covid-19 era. J NeuroInterv Surg.

[60] Diestro JDB, Li YM, Parra-Farinas C, Sarma D, Bharatha A, Marotta TR, Spears J (2020). Letter to the editor 'aneurysmal subarachnoid hemorrhage: collateral damage of COVID?'. World Neurosurg.

[61] Bernat AL, Giammattei L, Abbritti R, Froelich S. Impact of COVID-19 pandemic on subarachnoid hemorrhage. J Neurosurg Sci. 2020;64(4):409–410. 10.23736/S0390-5616.20.04963-2. 10.23736/S0390-5616.20.04963-2 32347681

[62] Kessler RA, Zimering J, Gilligan J, Rothrock R, McNeill I, Shrivastava RK, Caridi J, Bederson J, Hadjipanayis CG (2020). Neurosurgical management of brain and spine tumors in the COVID-19 era: an institutional experience from the epicenter of the pandemic. J Neurooncol.

[63] Bernhardt D, Wick W, Weiss SE, Sahgal A, Lo SS, Suh JH, Chang EL, Foote M, Perry J, Meyer B (2020). Neuro-oncology management during the COVID-19 pandemic with a focus on WHO grades III and IV gliomas. Neuro Oncol.

[64] Ostrom QT, Gittleman H, Truitt G, Boscia A, Kruchko C, Barnholtz-Sloan JS. CBTRUS Statistical report: primary brain and other central nervous system tumors diagnosed in the United States in 2011–2015. Neuro Oncol. 2018;20(suppl_4):iv1-iv86. 10.1093/neuonc/noy131. 10.1093/neuonc/noy131 PMC6129949 30445539

[65] de Joode K, Dumoulin DW, Tol J, Westgeest HM, Beerepoot LV, van den Berkmortel F, Mutsaers P, van Diemen NGJ, Visser OJ, Oomen-de Hoop E (2020). Dutch oncology COVID-19 consortium: outcome of COVID-19 in patients with cancer in a nationwide cohort study. Eur J Cancer.

[66] Lee AJX, Purshouse K (2021). COVID-19 and cancer registries: learning from the first peak of the SARS-CoV-2 pandemic. Br J Cancer.

[67] Ostrom QT, Cioffi G, Gittleman H, Patil N, Waite K, Kruchko C, Barnholtz-Sloan JS (2019). CBTRUS statistical report: primary brain and other central nervous system tumors diagnosed in the United States in 2012–2016. Neuro Oncol.

[68] Zou Y, Zhang J, Zhang T, Feng Y, Xiong Z, Xu C, Gong P, Si J, Chen J. Characteristics and operation outcomes of neuro-oncology patients after COVID-19 pandemic - a case series. Interdiscip Neurosurg. 2021;25:101172. 10.1016/j.inat.2021.101172. 10.1016/j.inat.2021.101172 PMC7955585 33754122

[69] Gu WT, Zhou F, Xie WQ, Wang S, Yao H, Liu YT, Gao L, Wu ZB (2021). A potential impact of SARS-CoV-2 on pituitary glands and pituitary neuroendocrine tumors. Endocrine.

[70] Troschel FM, Ahndorf F, Wille LM, Brandt R, Jost J, Rekowski S, Eich HT, Stummer W, Wiewrodt R, Jetschke K (2021). Quality of life in brain tumor patients and their relatives heavily depends on social support factors during the covid-19 pandemic. Cancers.

[71] Belani P, Schefflein J, Kihira S, Rigney B, Delman BN, Mahmoudi K, Mocco J, Majidi S, Yeckley J, Aggarwal A (2020). COVID-19 is an independent risk factor for acute ischemic stroke. AJNR Am J Neuroradiol.

[72] Al Saiegh F, Mouchtouris N, Khanna O, Baldassari M, Theofanis T, Ghosh R, Tjoumakaris S, Gooch MR, Herial N, Zarzour H (2021). Battle-tested guidelines and operational protocols for neurosurgical practice in times of a pandemic: lessons learned from COVID-19. World Neurosurg.

