# Supplementary material for: The impact of COVID-19 on clinical outcomes in people undergoing neurosurgery: a systematic review and meta-analysis
Source: Syst Rev. 2023 Aug 7;12:137. doi: 10.1186/s13643-023-02291-5 (PMC10405503; doi:10.1186/s13643-023-02291-5)

**SUPPLEMENTAL MATERIAL**

1. Table S1: Search strategies and results

2. Table S2: Risk of bias based on MINORS quality assessment

3. Figure S1: sensitivity analysis of the number of mortality

4. Figure S2: Forest plots for the number of mortality after excluding the article of Miękisiak et.al.

5. Figure S3: sensitivity analysis of the length of hospital stay

6. Figure S4: Forest plots for the length of stay after excluding the article of Bajunaid et.al.

**Table S1: Search strategies and results**

**Table S1: Search strategies and results**

**Database MEDLINE**

(("covid 19"[All Fields] OR "covid 19"[MeSH Terms] OR "covid 19 vaccines"[All Fields] OR "covid 19 vaccines"[MeSH Terms] OR "covid 19 serotherapy"[All Fields] OR "covid 19 nucleic acid testing"[All Fields] OR "covid 19 nucleic acid testing"[MeSH Terms] OR "covid 19 serological testing"[All Fields] OR "covid 19 serological testing"[MeSH Terms] OR "covid 19 testing"[All Fields] OR "covid 19 testing"[MeSH Terms] OR "sars cov 2"[All Fields] OR "sars cov 2"[MeSH Terms] OR "severe acute respiratory syndrome coronavirus 2"[All Fields] OR "ncov"[All Fields] OR "2019 ncov"[All Fields] OR (("coronavirus"[MeSH Terms] OR "coronavirus"[All Fields] OR "cov"[All Fields]) AND 2019/11/01:3000/12/31[Date - Publication]) OR ("sars cov 2"[MeSH Terms] OR "sars cov 2"[All Fields] OR "sars cov 2"[All Fields]) OR ("sars cov 2"[MeSH Terms] OR "sars cov 2"[All Fields] OR "2019 ncov"[All Fields])) AND ("neurosurgery"[MeSH Terms] OR "neurosurgery"[All Fields] OR "neurosurgeries"[All Fields] OR "neurosurgery s"[All Fields] OR "neurosurgical procedures"[MeSH Terms] OR ("neurosurgical"[All Fields] AND "procedures"[All Fields]) OR "neurosurgical procedures"[All Fields] OR ("neurosurgic"[All Fields] OR "neurosurgical"[All Fields] OR "neurosurgically"[All Fields])) AND ("cysts"[MeSH Terms] OR "cysts"[All Fields] OR "cyst"[All Fields] OR "neurofibroma"[MeSH Terms] OR "neurofibroma"[All Fields] OR "neurofibromas"[All Fields] OR "tumor s"[All Fields] OR "tumoral"[All Fields] OR "tumorous"[All Fields] OR "tumour"[All Fields] OR "neoplasms"[MeSH Terms] OR "neoplasms"[All Fields] OR "tumor"[All Fields] OR "tumour s"[All Fields] OR "tumoural"[All Fields] OR "tumourous"[All Fields] OR "tumours"[All Fields] OR "tumors"[All Fields] OR ("astrocytoma"[MeSH Terms] OR "astrocytoma"[All Fields] OR "astrocytomas"[All Fields]) OR ("ependymoma"[MeSH Terms] OR "ependymoma"[All Fields] OR "ependymomas"[All Fields]) OR ("glioma"[MeSH Terms] OR "glioma"[All Fields] OR "gliomas"[All Fields] OR "glioma s"[All Fields]) OR ("glioblastoma"[MeSH Terms] OR "glioblastoma"[All Fields] OR "glioblastomas"[All Fields]) OR ("meningioma"[MeSH Terms] OR "meningioma"[All Fields] OR "meningiomas"[All Fields]) OR ("aneurysm"[MeSH Terms] OR "aneurysm"[All Fields] OR "aneurysms"[All Fields] OR "aneurysm s"[All Fields] OR "aneurysmal"[All Fields] OR "aneurysmally"[All Fields] OR "aneurysmic"[All Fields]) OR ("intracranial haemorrhage"[All Fields] OR "intracranial hemorrhages"[MeSH Terms] OR ("intracranial"[All Fields] AND "hemorrhages"[All Fields]) OR "intracranial hemorrhages"[All Fields] OR ("intracranial"[All Fields] AND "hemorrhage"[All Fields]) OR "intracranial hemorrhage"[All Fields]))) AND (y_5[Filter])

| **Table S2 Risk of bias based on MINORS quality assessment** | | | | | | | | |  |  |
| --- | --- | --- | --- | --- | --- | --- | --- | --- | --- | --- |
| NO | item | Amarouche2021 | Amoo  2021 | Ashkan  2021 | Bajunaid2020 | Fiorindi  2022 | Han  2021 | Kashefiolasl2022 | Mallari  2021 | Miękisiak  2022 |
| 1 | A clearly stated aim | 2 | 2 | 2 | 2 | 2 | 2 | 2 | 2 | 2 |
| 2 | Inclusion of consecutive patients | 2 | 2 | 2 | 2 | 2 | 2 | 2 | 2 | 2 |
| 3 | Prospective collection of data | 0 | 0 | 2 | 0 | 0 | 0 | 0 | 0 | 0 |
| 4 | Endpoints appropriate to the aim of the study | 2 | 2 | 2 | 2 | 2 | 2 | 2 | 1 | 2 |
| 5 | Unbiased assessment of the study endpoint | 0 | 1 | 1 | 0 | 1 | 0 | 1 | 1 | 1 |
| 6 | Follow-up period appropriate to the aim of the study | 2 | 2 | 2 | 2 | 2 | 2 | 2 | 2 | 2 |
| 7 | Loss to follow up less than 5% | 2 | 2 | 2 | 2 | 2 | 2 | 2 | 2 | 2 |
| 8 | Prospective calculation of the study size | 0 | 0 | 1 | 0 | 0 | 0 | 0 | 0 | 0 |
| 9 | An adequate control group | 2 | 2 | 2 | 2 | 2 | 2 | 2 | 2 | 2 |
| 10 | Contemporary groups | 0 | 0 | 0 | 0 | 0 | 0 | 0 | 0 | 0 |
| 11 | Baseline equivalence of groups | 2 | 2 | 2 | 2 | 2 | 2 | 2 | 2 | 2 |
| 12 | Adequate statistical analyses | 2 | 2 | 2 | 2 | 2 | 2 | 2 | 2 | 2 |
| 13 | Total score | 16 | 17 | 20 | 16 | 17 | 16 | 17 | 16 | 17 |
| The items are scored 0 (not reported), 1 (reported but inadequate) or 2 (reported and adequate). The global ideal score being 16 for non-comparative studies and 24 for comparative studies. | | | | | | | | |  |  |

| **Table S2 Risk of bias based on MINORS quality assessment** | | | | | | | | |
| --- | --- | --- | --- | --- | --- | --- | --- | --- |
| NO | item | Norman  2021 | Qureshi  2022 | Qureshi  2021 | Theofanopoulos  2021 |  |  |  |
| 1 | A clearly stated aim | 2 | 2 | 2 | 2 |  |  |  |
| 2 | Inclusion of consecutive patients | 2 | 2 | 2 | 2 |  |  |  |
| 3 | Prospective collection of data | 0 | 0 | 0 | 0 |  |  |  |
| 4 | Endpoints appropriate to the aim of the study | 2 | 2 | 2 | 2 |  |  |  |
| 5 | Unbiased assessment of the study endpoint | 1 | 1 | 1 | 0 |  |  |  |
| 6 | Follow-up period appropriate to the aim of the study | 2 | 2 | 2 | 2 |  |  |  |
| 7 | Loss to follow up less than 5% | 2 | 2 | 2 | 2 |  |  |  |
| 8 | Prospective calculation of the study size | 0 | 0 | 0 | 0 |  |  |  |
| 9 | An adequate control group | 2 | 2 | 2 | 2 |  |  |  |
| 10 | Contemporary groups | 0 | 0 | 0 | 0 |  |  |  |
| 11 | Baseline equivalence of groups | 2 | 2 | 2 | 2 |  |  |  |
| 12 | Adequate statistical analyses | 2 | 2 | 2 | 2 |  |  |  |
| 13 | Total score | 15 | 17 | 17 | 16 |  |  |  |
| The items are scored 0 (not reported), 1 (reported but inadequate) or 2 (reported and adequate). The global ideal score being 16 for non-comparative studies and 24 for comparative studies. | | | | | | | | |

Figure S1. sensitivity analysis of the number of mortality


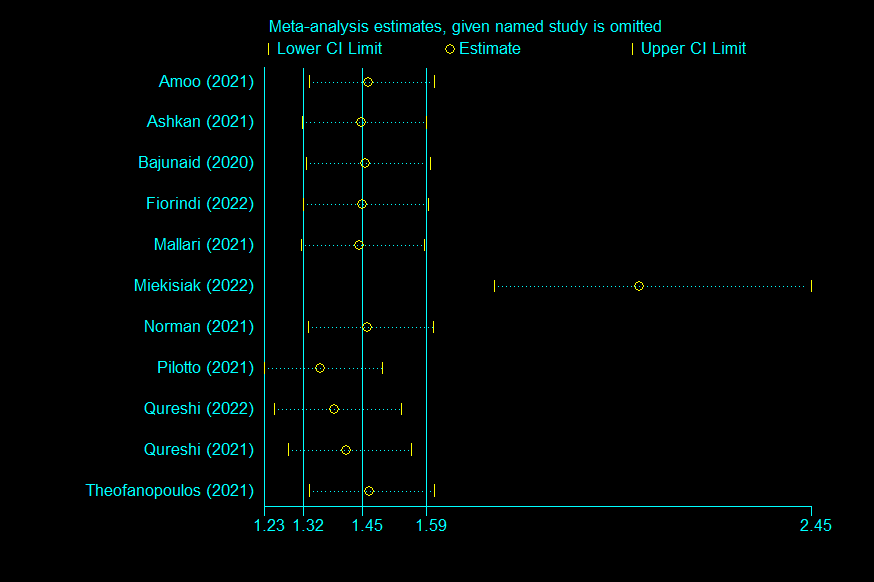


Figure S2: Forest plots for the number of mortality after excluding the article of Miękisiak et.al.


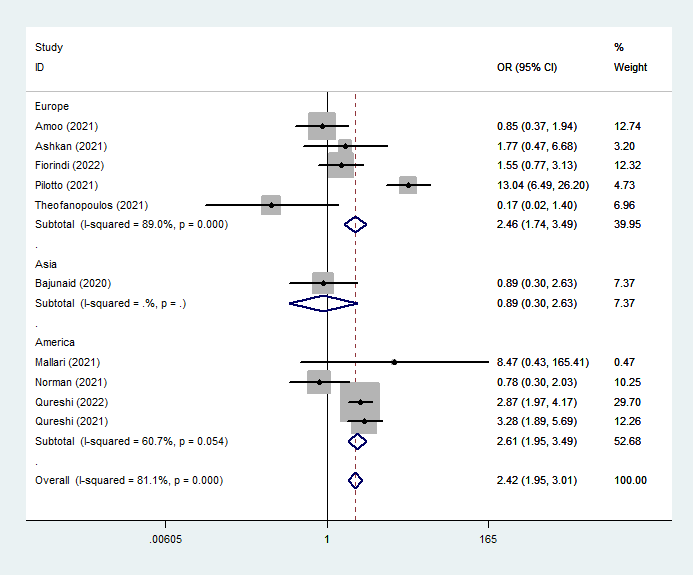


Figure S3: sensitivity analysis of the length of hospital stay


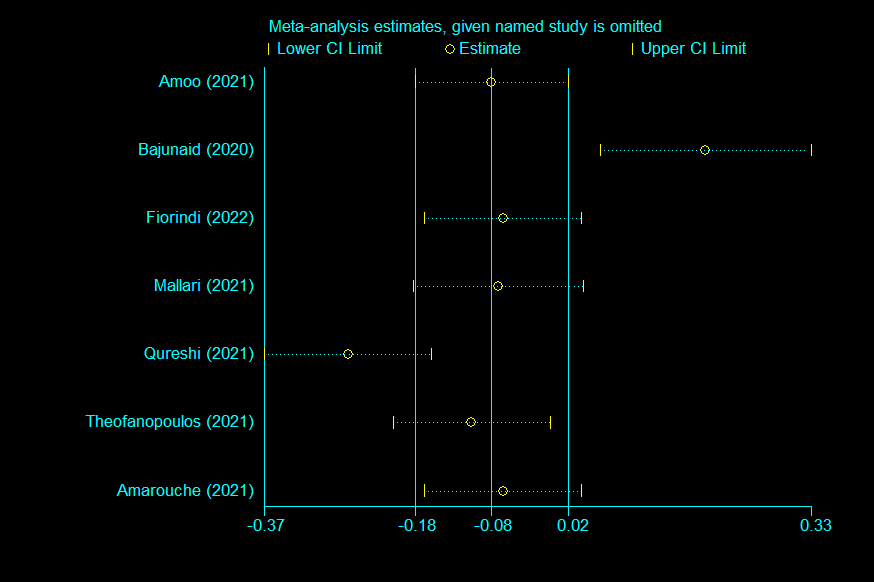


Figure S4: Forest plots for the length of stay after excluding the article of Bajunaid et.al.


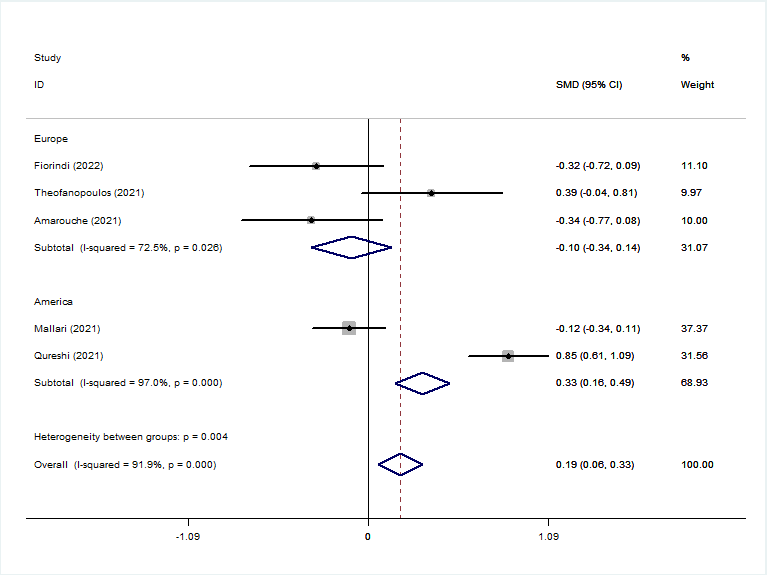

Supplement: Supplementary file 1 — Additional file 1: Table S1. Search strategies and results. Table S2. Risk of bias based on MINORS quality assessment. Figure S1. Sensitivity analysis of the number of mortality. Figure S2. Forest plots for the number of mortality after excluding the article of Miękisiak et.al. Figure S3. sensitivity analysis of the length of hospital stay. Figure S4. Forest plots for the length of stay after excluding the article of Bajunaid et al. [file 13643_2023_2291_MOESM1_ESM.docx]
